# Combination of poly I:C and Pam3CSK4 enhances activation of B cells *in vitro* and boosts antibody responses to protein vaccines *in vivo*

**DOI:** 10.1371/journal.pone.0180073

**Published:** 2017-06-29

**Authors:** Genevieve M. Weir, Mohan Karkada, David Hoskin, Marianne M. Stanford, Lisa MacDonald, Marc Mansour, Robert S. Liwski

**Affiliations:** 1 Research & Development, Immunovaccine Inc, Halifax, Nova Scotia, Canada; 2 Department of Microbiology & Immunology, Dalhousie University, Halifax, Nova Scotia, Canada; 3 Department of Pathology, Dalhousie University, Halifax, Nova Scotia, Canada; 4 Department of Pathology and Laboratory Medicine, Division of Hematopathology, Queen Elizabeth II Health Sciences Centre, Halifax, Nova Scotia, Canada; INSERM, FRANCE

## Abstract

Vaccines that can rapidly induce strong and robust antibody-mediated immunity could improve protection from certain infectious diseases for which current vaccine formulations are inefficient. For indications such as anthrax and influenza, antibody production *in vivo* is a correlate of efficacy. Toll-like receptor (TLR) agonists are frequently studied for their role as vaccine adjuvants, largely because of their ability to enhance initiation of immune responses to antigens by activating dendritic cells. However, TLRs are also expressed on B cells and may contribute to effective B cell activation and promote differentiation into antigen-specific antibody producing plasma cells *in vivo*. We sought to discover an adjuvant system that could be used to augment antibody responses to influenza and anthrax vaccines. We first characterized an adjuvant system *in vitro* which consisted of two TLR ligands, poly I:C (TLR3) and Pam3CSK4 (TLR2), by evaluating its effects on B cell activation. Each agonist enhanced B cell activation through increased expression of surface receptors, cytokine secretion and proliferation. However, when B cells were stimulated with poly I:C and Pam3CSK4 in combination, further enhancement to cell activation was observed. Using B cells isolated from knockout mice we confirmed that poly I:C and Pam3CSK4 were signaling through TLR3 and TLR2, respectively. B cells activated with Poly I:C and Pam3CSK4 displayed enhanced capacity to stimulate allogeneic CD4^+^ T cell activation and differentiate into antibody-producing plasma cells *in vitro*. Mice vaccinated with influenza or anthrax antigens formulated with poly I:C and Pam3CSK4 in DepoVax^™^ vaccine platform developed a rapid and strong antigen-specific serum antibody titer that persisted for at least 12 weeks after a single immunization. These results demonstrate that combinations of TLR adjuvants promote more effective B cell activation *in vitro* and can be used to augment antibody responses to vaccines *in vivo*.

## Introduction

Pre-existing antibodies offer the best protection against infection, and many vaccines are available that can effectively mitigate risk of serious infection through prophylactic immunizations. However, for some indications current vaccine formulations do not provide adequate protection. Hence, there is an urgent need to develop novel vaccine technologies to meet these needs. Influenza and anthrax are two examples of indications for which improved and/ or new vaccines are required. Although these indications are quite different in terms of pathogenesis and antigen type, antibody production is the correlate of a protective immune response for both. Therefore, an optimal vaccine for these infectious diseases would induce a rapid and long lasting antibody mediated response with minimal immunizations [1, 2].

A vaccine platform called DepoVax^™^ (DPX) is a unique water-free formulation that generates strong, long lasting immune responses. DPX is a lipid-in-oil formulation that can incorporate a variety of different peptide or protein antigens. Upon injection, DPX creates a depot that facilitates active uptake by antigen-presenting cells [3]. DPX is a fully characterized formulation, and DPX formulations containing MHC class I restricted peptides have demonstrated robust cellular immune responses when tested in phase I clinical oncology indications [4, 5]. In preclinical models, DPX formulations have shown enhanced antibody mediated immune responses and have supported an ongoing phase I trial of a DPX vaccine for respiratory syncytial virus. The ability of DPX to generate rapid and long-lasting immune responses following a single immunization makes it particularly suitable for development of influenza and anthrax vaccines [6, 7].

The type of immune response elicited by DPX can be tailored by changing the adjuvants used in the formulation. Adjuvants influence the type and strength of immune response towards vaccination primarily through activating the innate immune response, which in turn activates the adaptive immune response [8–10]. Toll-like receptors (TLRs) are primarily found on innate immune cells and are common targets of adjuvants [11]; TLRs are also expressed by naïve B cells and TLR stimulation can influence their differentiation into antibody-secreting plasma cells (ASCs) [12]. Unlike T cells, B cells recognize intact soluble antigen without requiring MHC presentation [13, 14]. The transition of naïve B cells into ASC is completed when CD4^+^ T cells recognize cognate peptide presented in MHC II by B cells. CD4^+^ T cells provide co-stimulation through CD40-CD40L interaction and cytokines to induce antigen class switching. Similar to innate antigen-presenting cells, naïve B cells also express several TLRs [15, 16]. TLR stimulation during B cell activation influences the maturation process by skewing class switch recombination, robustness of antibody production, cytokine secretion and the function of B cells as antigen presenting cells [17–20].

The effects of TLR stimulation on B cells has largely been investigated using LPS (stimulating TLR4) or CpG (stimulating TLR9) since receptors are the most abundant TLRs on the surface of naïve B cells [12, 21]. However, B cells express other TLRs at lower levels which may have different effects on B cell differentiation. Indeed, combinations of two of more TLR ligands have been reported to act synergistically to enhance activation of a variety of innate immune cells *in vitro* [22, 23] and boost responses to vaccination *in vivo* [24]. There is limited information on the effect of stimulating B cells with multiple TLR ligands *in vitro* and the use of TLR agonist combinations to enhance antibody responses *in vivo*. TLR3 and TLR1/2 are expressed at low levels on naïve B cells, yet B cells respond to stimulation to their respective ligands, poly I:C and Pam3CSK4 *in vitro* [25, 26]. Poly I:C and Pam3CSK4 have been used as adjuvants to boost antibody responses to vaccines *in vivo*, in some cases to a greater extent than CpG [27, 28]. Combined poly I:C and Pam3CSK4 stimulation has been reported to synergistically enhance activation of dendritic cells and macrophages *in vitro* [29, 30] which may be due in part to the different signaling pathways used by each receptor [8]. In this paper we have sought to identify a unique adjuvant system that may be used to boost antibody responses to DPX vaccines targeting influenza hemagglutinin (HA) H5N1 antigen and anthrax protective antigen (PA). Therefore, we studied whether the combination of poly I:C and Pam3CSK4 stimulation could enhance activation of B cells *in vitro*, and whether this combination could augment antibody production to influenza and anthrax DPX vaccines *in vivo*.

## Materials and methods

### Animals

Female BALB/c, C57BL/6 and CD-1 mice (4–6 weeks old) were obtained from Charles River Labs (St. Constant, PQ). TLR2 knockout mice had a C57BL/6 background and were a kind gift from Dr. Jean Marshall (Dalhousie University, NS, Canada). TLR3 knockout mice (B6;129S1-Tlr3tm1Flv/J) and wild type controls (B6;129SF2/J) were obtained from Jackson Laboratories (Bar Harbor, ME). Mice were housed under filter-top conditions and provided food and water *ad libitum*. Institutional animal care and use guidelines set by Carleton Animal Care Facility at Dalhousie University (Halifax, NS, Canada) were followed for all experiments. All experimental procedures performed during this study were approved by the Ethics Committee at Dalhousie University and strictly followed the guidelines set by The Canadian Council on Animal Care.

### TLR agonists

Poly I:C was obtained from Thermo Fisher Scientific (Milwaukee, WS). Pam3CSK4 was obtained from EMC Microcollections (Tuebingen, Germany). Lipopolysaccharide (LPS) and CpG 1826 were purchased from Sigma-Aldrich (St. Louis, MO). All agonists were reconstituted in water at the concentration recommended by manufacturer; for cell stimulation, appropriate dilutions were prepared in complete medium.

### B cell purification and culture

B cells were isolated from naïve mouse spleens by negative selection (Stem Cell Technologies, Vancouver, BC). The purified B cells used for all experiments were phenotypically characterized as >95% CD19^+^B220^+^IgD^+^CD23^-/low^CD5^-^CD11c^-^, indicating that they were predominantly B-2 marginal zone B cells [26] (S1 Fig). Purified B cells were cultured in triplicate in 96-well plates at 10^5^ cells/ well in RPMI 1640 medium supplemented with 10% fetal calf serum (FCS; HyClone, Rockford, IL), 2% penicillin-streptomycin (Gibco, Burlington, ON, Canada), 50 mM mercaptoethanol (Gibco) and 2 mM L-glutamine (Gibco). Pam3CSK4 and poly I:C dilutions were made in complete medium and added into the wells containing B cells for a final volume of 200 μL per well. T-cell-dependent B cell activation was simulated by adding purified hamster anti-mouse CD40 (2.5 μg/mL; clone HM40-3, BD Biosciences, Mississauga, ON) and purified rat anti-mouse kappa Ig (1 μg/mL; clone 187.1, BD Biosciences) to the B cell suspensions. Plates were incubated at 37°C/ 5% CO_2_.

### Proliferation of B cells

To measure proliferation, B cells were incubated for 3 days and pulsed with 0.5 μCi of tritiated thymidine ([^3^H]-TdR; MP Biomedical, Irvine, CA) for the last 18 hours of culture. Cells were harvested onto fiberglass filter mats (Skatron Instruments, Sterling, VA) with a Titertek Cell Harvester (Skatron Instruments). [^3^H]-TdR uptake was assessed using a Beckman LS6000IC liquid scintillation counter (Beckman Coulter Inc., Mississauga, ON) and quantified as counts per minute (CPM). The values of each experiment were taken from the average of triplicate wells.

### Flow cytometry

For measurement of surface receptor expression, B cells were harvested after 24 hours of culture for flow cytometry. All antibodies used for flow cytometry were purchased from eBioscience (San Diego, CA) unless otherwise stated. After blocking with purified anti-CD16/CD32 (clone 93), cells were stained using cocktails of: CD19-APC (clone 1D3), CD80-FITC (clone 16-10A1), CD86-PE (clone GL1), CD25-APC (clone PC61), MHC class II-APC (clone M5/114.15.2), CD40-PE (clone 1C10), CD69-FITC (H1.2F3), CD138-APC (clone 281–2; BD Bioscience), CD267-PE (TACI; clone ebio8F10-3). Cells were acquired using a FACSCalibur^®^ (BD Bioscience) and data was analyzed using WinList 7.0 software (Verity Software, Topsham, ME).

### Quantitation of cytokine and immunoglobulin production

Cytokines were detected in B cell supernatants harvested after 24 hours. Commercially available ELISA kits from eBioscience were used for IL-2, IL-4, IL-6, IL-10, IL-12p70, IFN-γ, and CXCL10. A cytokine bead array (CBA) kit from BD Bioscience was used to measure TNF-α and IL-21 production. IgG production was measured in supernatants harvested after 4 days of culture using a commercially available ELISA kit from eBioscience.

### Mixed lymphocyte reaction

Purified B cells were stimulated with poly I:C and/ or Pam3CSK4, as well as anti-CD40 and anti-Ig, as described above. After 24 hours, B cells were harvested and treated with 50 μg/mL of mitomycin C (Sigma) for 1 hour at 37°C. B cells were then washed three times in supplemented RPMI medium, counted and resuspended at 10^5^ cells/mL, 4×10^4^ cells/mL and 2×10^4^ cells/mL. Each dilution of B cell was added to a 96-well plate in triplicate (100 μL/ well). CD4^+^ T cells were purified from spleens of BALB/c mice using negative magnetic separation (Stemcell Technologies) and resuspended at 10^6^ cells/mL. T cells were added to wells containing B cells (100 μL/ well) and co-cultures were incubated for 3 days. Proliferation and flow cytometric analysis was performed after 3 days of culture as described above. CD4^+^ T cells were identified using anti-CD4 (clone GK1.5).

### Vaccines and immunization

DepoVax^™^ vaccines were prepared as previously described [31, 32]. Briefly, proteins and adjuvants were solubilized in appropriate buffer and mixed with S100 lipids and cholesterol (both from Lipoid GmBH, Germany). The aqueous mixture was lyophilized to a dry cake which was reconstituted with Montanide ISA51 VG (SEPPIC, France) just prior to injection. Vaccines were administered via intramuscular vaccination 25 μL on each the right and left hind legs. Influenza vaccine was prepared with recombinant hemagglutinin protein (rHA; Protein Sciences, Meriden, CT) to deliver 0.5 μg antigen, 1 μg poly I:C, and/ or 1 μg Pam3CSK4 in a 50 μL dose. Anthrax vaccine was prepared with recombinant protective antigen (rPA; List Biologicals, Campbell, CA) to deliver 1 μg antigen, 1 μg poly I:C, and/ or 1 μg Pam3CSK4 per 50 μL dose.

### Antibody endpoint titration

Serum samples were collected via facial venupuncture at 4, 8, 12 and 16 weeks post immunization. Endpoint antibody titers were determined by ELISA. Briefly, plates were coated overnight with1 μg/mL of either rHA or rPA antigen. After blocking with 3% gelatin, serial dilutions of the serum were applied and plates were incubated overnight at 4°C. Next day, bound antibody was detected using an alkaline phosphatase-linked Protein A (Calbiochem, Gibbstown, NJ) as the secondary reagent. Endpoint titers were defined as the reciprocal of the highest dilution above the cutoff value; cutoff values were calculated using a 95% confidence interval [33].

### Statistical analysis

Statistical analysis was conducted with GraphPad Prism 5 software (La Jolla, CA). Data was analysed by one-way ANOVA using Tukey post-test, two-way ANOVA with Bonferroni multiple comparisons test, or Student’s t-test as indicated; *p≤0.05, **p≤0.01, ***p≤0.001. For the in vitro experiments, data is shown as the average of multiple individual B cell preparations which were assayed in groups of 1–3 in independent experiments as indicated in figure legends.

## Results

### Poly I:C and Pam3CSK4 promote B cell activation independent of T cell help

**Fig 1:**
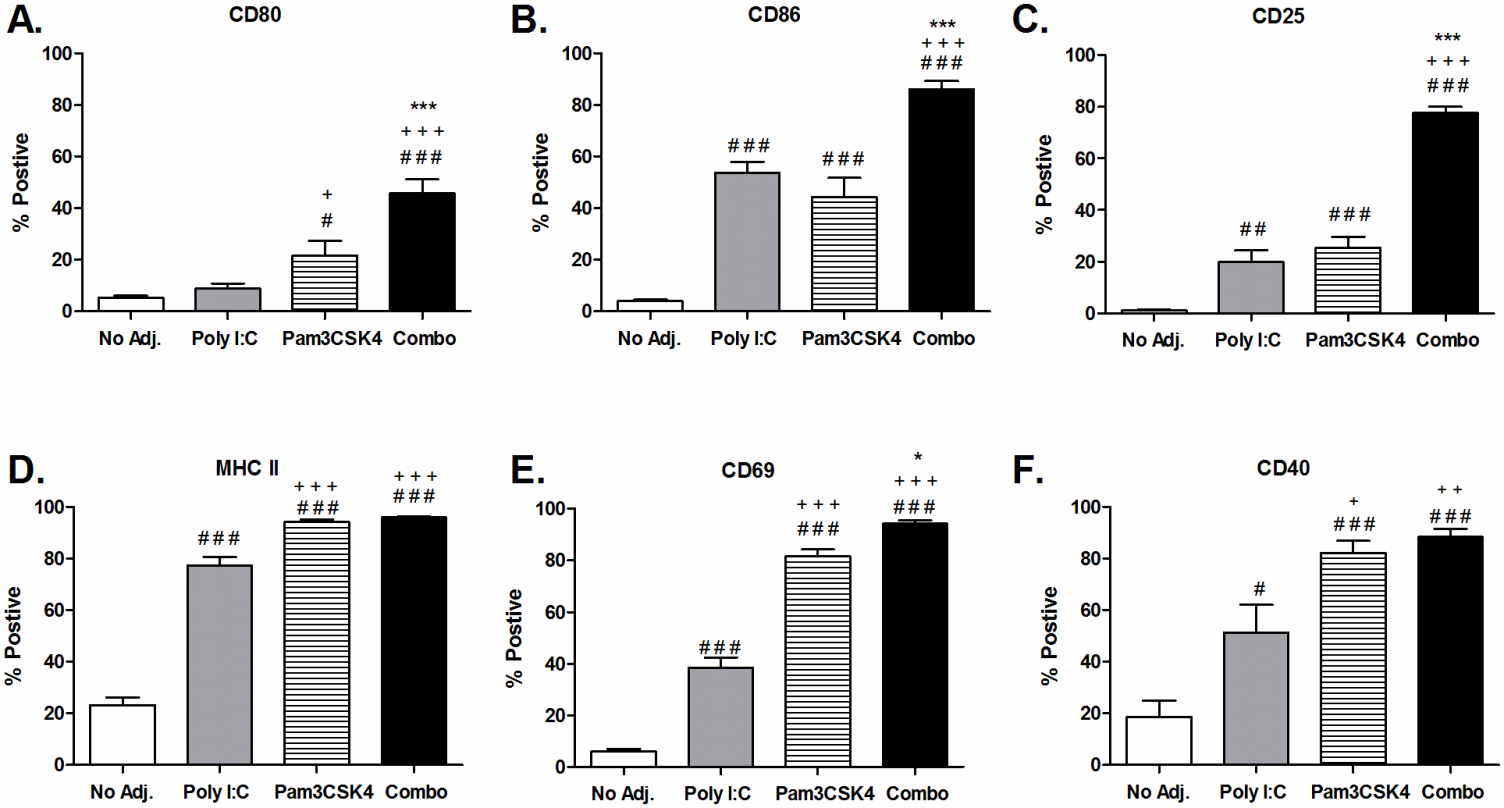
Poly I:C and Pam3CSK4 stimulate expression of surface receptors in B cells independent of T cell help. CD19^+^ B cells were purified from spleens of C57BL6 mice and stimulated with poly I:C (25 μg/mL), Pam3CSK4 (1 μg/mL), or the combination of both. Expression of surface receptor was determined after 24 hours by flow cytometry. (A) CD80 (n = 11), (B) CD86 (n = 7), (C) CD25 (n = 6), (D) MHC class II (n = 5), (E) CD69 (n = 4), (F) CD40 (n = 6). Data are shown as average ± SEM of individual B cell preparations as indicated, data is pooled from two to four independent experiments. Statistics performed by ANOVA with Tukey post-test: “#” indicates significance relative to untreated, “+” indicates significance relative to poly I:C and “*” indicates significance relative to Pam3CSK4.

Different TLR agonists have been reported to selectively increase activation markers on B cells after 24 hour stimulation [25, 26]. In a preliminary experiment, we dosed poly I:C and Pam3CSK4 separately (S3 Fig) and in combination (S4 Fig) to identify the optimal dose of each agonist that could result in highest combined effect. Poly I:C was tested in a range of 0.1 to 50 ug/mL and Pam3CSK4 in a range of 0.01 to 10 ug/mL. Activation was assessed by expression of receptors associated with B cell activation, CD80 and CD40. We selected the dose of 25 μg/mL for poly I:C and 1 μg/mL for Pam3CSK4 as having a suboptimal effect on the induction of these receptors when used individually, but resulted in very high expression when used in combination. We then tested the agonists at these doses alone and in combination to see if they could augment expression of a more comprehensive set of markers: CD80, CD86, CD25, MHC class II, CD69 and CD40 (Fig 1; S3 Fig). We found that each agonist alone induced a unique profile of receptors in the B cells. In general, poly I:C induced lower levels of receptor expression than Pam3CSK4. The receptors CD80, CD86, CD25 and CD69 were significantly increased by combination therapy compared to each poly I:C and Pam3CSK4 treatments. CD40 and MHC class II were substantially increased by Pam3CSK4 alone treatment, which was not augmented further by combination treatment.

### Poly I:C and Pam3CSK4 enhance T-cell-dependent activation of B cells

**Fig 2:**
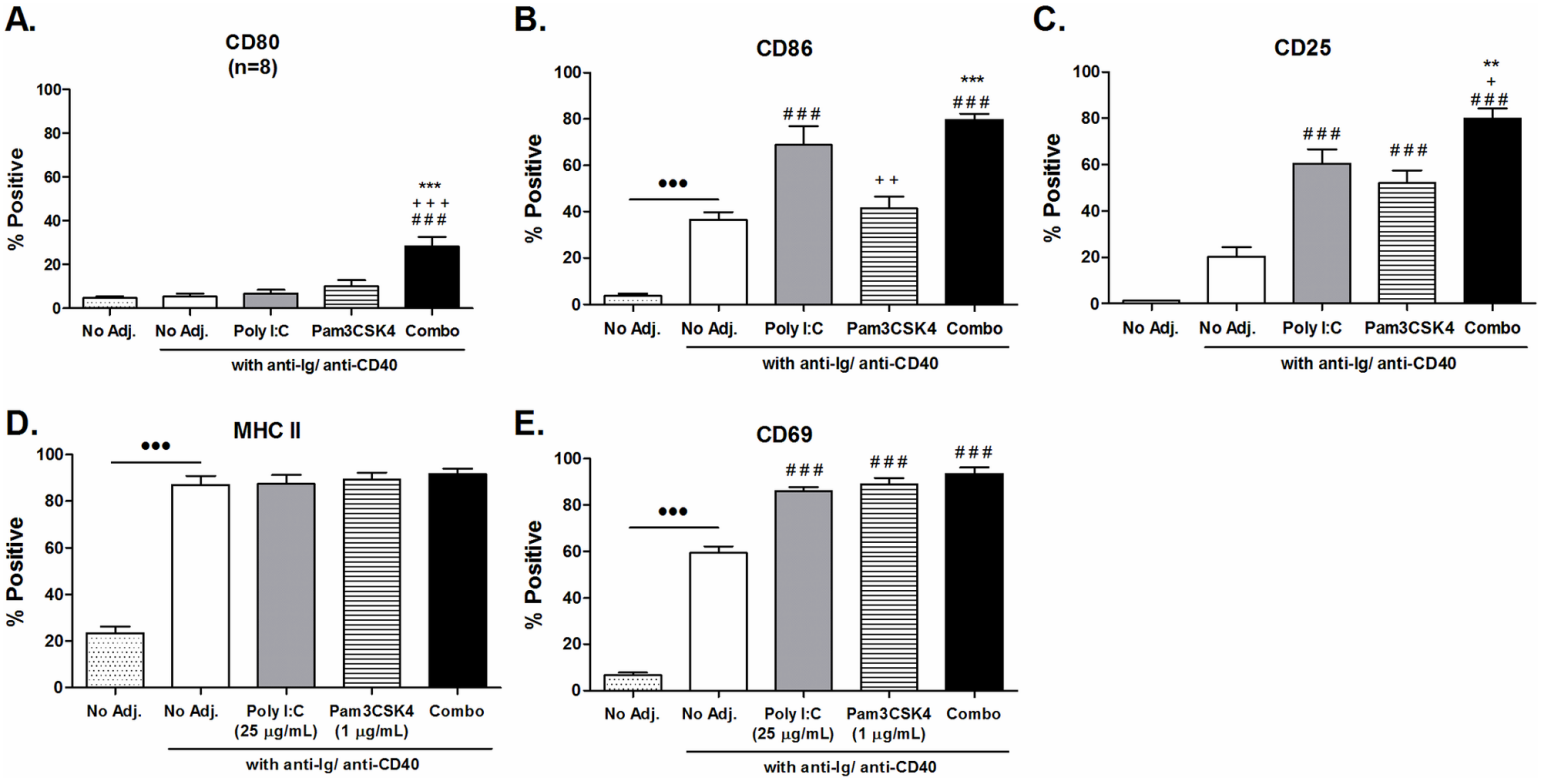
Poly I:C and Pam3CSK4 enhance T-cell-dependent B cell activation. Purified B cells from C57BL/6 mice were stimulated with poly I:C (25 μg/mL), Pam3CSK4 (1 μg/mL) or a combination of both, with T-cell-dependent co-stimulation provided by anti-CD40 and anti-Ig. Expression of surface receptor was determined after 24 hours by flow cytometry. (A) CD80 (n = 8), (B) CD86 (n = 5), (C) CD25 (n = 6), (D) MHC class II (n = 7) and (E) CD69 (n = 3). Data are shown as average ± SEM of individual B cell preparations as indicated, data is pooled from two to four independent experiments. Statistics performed by ANOVA with Tukey post-test: “#” indicates significance relative to untreated, “+” indicates significance relative to poly I:C and “*” indicates significance relative to Pam3CSK4.

TLR stimulation has been reported to synergize with T-cell-dependent B cell activation *in vitro* [15, 16], and is more representative of B cell activation *in vivo*. Therefore, we tested the effects of poly I:C and/ or Pam3CSK4 stimulation of B cells activated in a T-cell-dependent manner by simulating T-cell-dependent activation with the addition of anti-Ig and anti-CD40 into cultures. After 24 hour incubation, we looked at the expression of surface receptors. The T-cell-dependent activation alone induced higher baseline expression of CD86, MHC class II and CD69 (Fig 2B, 2D and 2E), but no significant increase in CD80 or CD25 (Fig 2A and 2C). T-cell-dependent activation augmented the expression of CD25 and CD69 in response to stimulation with poly I:C or Pam3CSK4 alone, and overall the combination of both adjuvants resulted in expression levels similar to that achieved without T-cell-dependent activation (Fig 1).T-cell-dependent T-cell-dependent The effects on CD40 expression could not be reliably detected due to the blocking effect of the anti-CD40 antibody used for T-cell-dependent activation.

**Fig 3:**
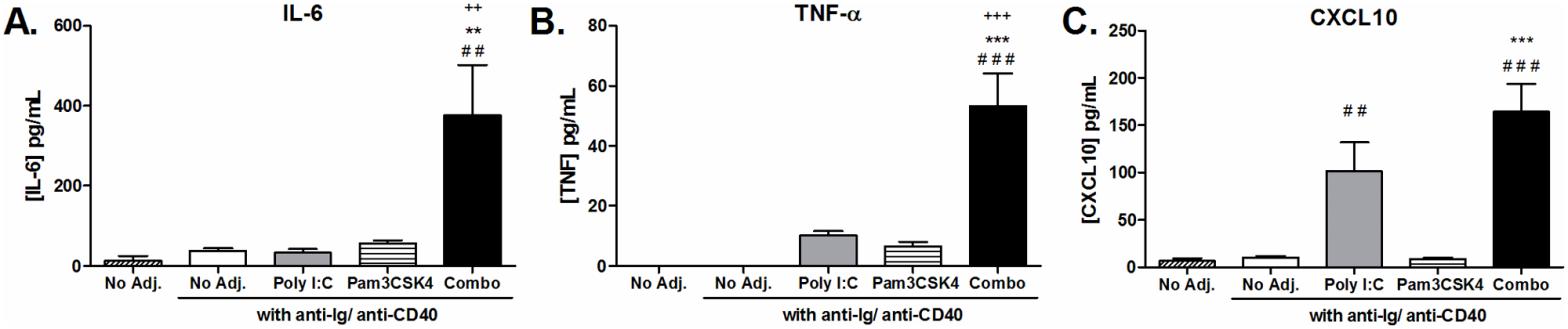
Cytokine production enhanced by poly I:C and Pam3CSK4 stimulated B cells. Purified B cells from C57BL/6 mice were stimulated with poly I:C (25 μg/mL), Pam3CSK4 (1 μg/mL) or a combination of both, with T-cell-dependent co-stimulation provided by anti-CD40 and anti-Ig. Supernatants were harvested after 24 hours and levels of (A) IL-6 (n = 6), (B) TNF-α (n = 6) and (C) CXCL10 (n = 8) determined by ELISA or CBA analysis. Data are shown as average ± SEM of individual B cell preparations as indicated, data is pooled from three to five independent experiments. Statistics performed by ANOVA with Tukey post-test: “#” indicates significance relative to untreated, “+” indicates significance relative to poly I:C and “*” indicates significance relative to Pam3CSK4.

Stimulation of B cells with TLR agonists has been reported to increase production of several cytokines [25, 34]. We screened for production of cytokines important to B cell survival and proliferation: IL-6, IL-10, IL-21 and TNFα; Th1 cytokines: IL-12p70 and IFN-γ; and Th2 cytokine: IL-4. Since poly I:C is also known to induce CXCL10 production in other cell types [35] and CpG stimulation of B cells induces CXCL10 [36], we also tested for production of the chemokine CXCL10. We could only detect production of IL-6, TNFα and CXCL10 (Fig 3) as the remaining cytokines were below the limits of detection. The combination of poly I:C and Pam3CSK4 significantly enhanced production of IL-6 and TNFα above no agonist or single agonist stimulation (Fig 3A and 3B). Poly I:C alone induced significant production of the chemokine CXCL10, and in combination with Pam3CSK4 this level was not increased further (Fig 3C).

**Fig 4:**
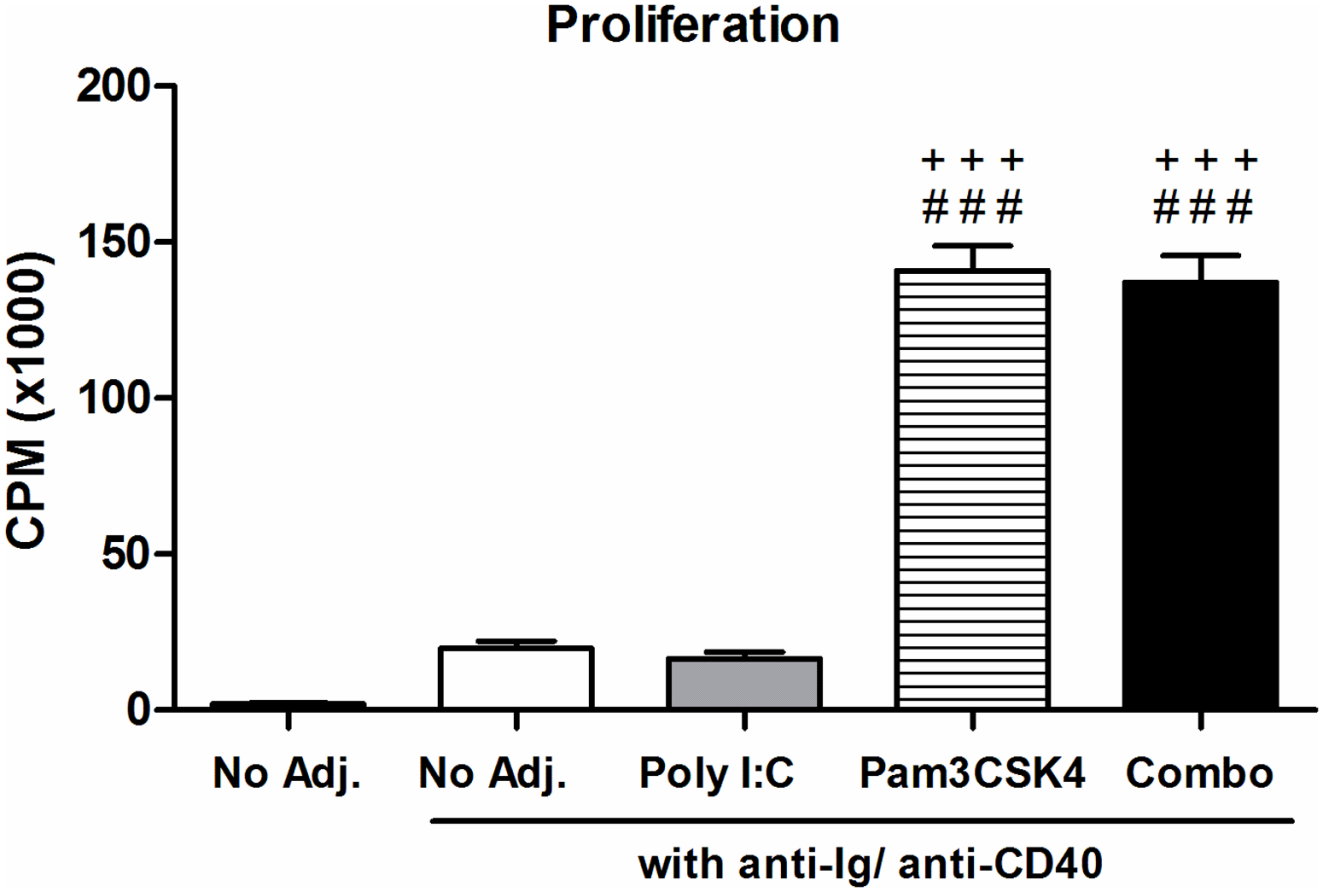
Proliferative response of B cells stimulated with poly I:C and Pam3CSK. Purified B cells from C57BL/6 mice were stimulated with poly I:C (25 μg/mL), Pam3CSK4 (1 μg/mL) or a combination of both, with T-cell-dependent co-stimulation provided by anti-CD40 and anti-Ig. Proliferation was measured after 3 days by [^3^H]-TdR uptake. Data are shown as average ± SEM of 5 individual B cell preparations, data is pooled from at two independent experiments. Statistics performed by ANOVA with Tukey post-test: “#” indicates significance relative to untreated, “+” indicates significance relative to poly I:C and “*” indicates significance relative to Pam3CSK4.

Finally, we measured how the proliferation of B cells with T-cell-dependent activation was affected by the agonists (Fig 4). After 3 days of culture, Pam3CSK4 induced a significant increase in B cell proliferation alone, which was not increased further with poly I:C combination.

### TLR3 and TLR2 are essential to optimal B cell activation by poly I:C and Pam3CSK4

**Fig 5:**
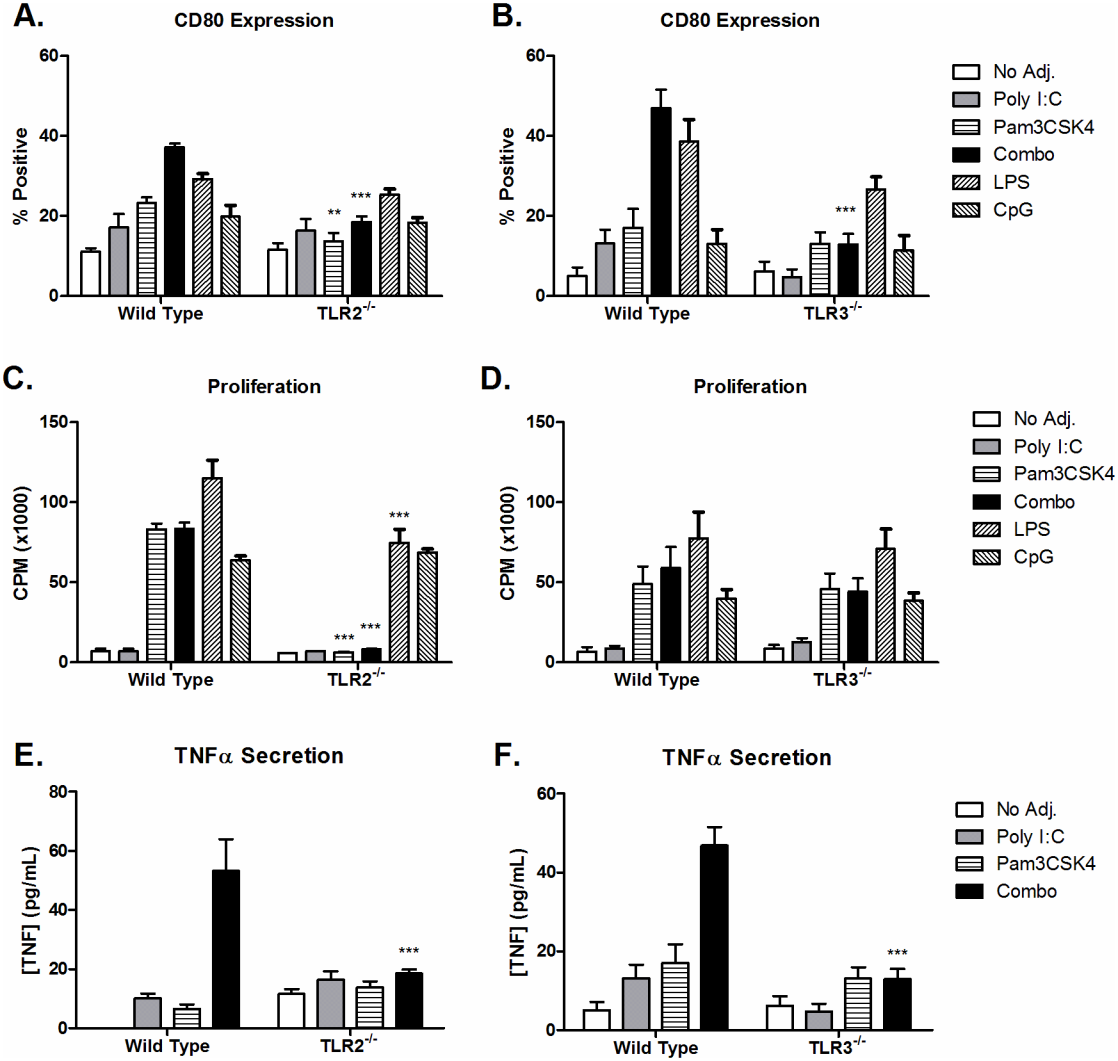
TLR3 and TLR2 Mediate B cell Response to poly I:C and Pam3CSK4 respectively. B cells purified from TLR2^-/-^ (A, C, E) or TLR3^-/-^ (B, D, F) mice were stimulated with poly I:C (25 μg/mL), Pam3CSK4 (1 μg/mL), the combination of poly I:C and Pam3CSK4, LPS (10 μg/mL) or CpG (25 μg/mL), with T-cell-dependent co-stimulation provided by anti-CD40 and anti-Ig. Relevant wild-type controls were tested in parallel (C57BL/6 for TLR2^-/-^, B6;129SF2/J for TLR3^-/-^). (A, B) Expression of CD80 was measured by flow cytometry after 24 hours. (C, D) Proliferation was measured by [^3^H]-TdR uptake after 3 days. (E, F) Production of TNF-α was measured by ELISA the supernatants harvested at 24 hours. Results are shown as the average ± SEM; TLR2^-/-^ is from 4 individual B cell preparations pooled from two independent experiments; TLR3^-/-^ is from 5 individual B cell preparations pooled from three independent experiments. Statistics compare the response of knockout B cells to corresponding wild type B cells and were calculated by 2-way ANOVA with Bonferroni post-test, *p<0.05, ***p<0.001.

To confirm that poly I:C and Pam3CSK4 were mediating effects through the TLR pathway, we performed experiments using B cells isolated from TLR3 and TLR2 knockout mice. This was important in particular for poly I:C since two other receptors have been reported to recognize this agonist, MDA-5 and RIG-I [9]. It was also of interest to us to see if these effects may be due to an alternate receptor, given that TLR expression on B cells has been reported to be low [25, 26]. Using methods already described, we evaluated stimulation of B cells isolated from TLR2-/- and TLR3-/- knockout mice with poly I:C and Pam3CSK4 (Fig 5). Wild-type B cells were stimulated in parallel.

In comparison to wild type B cells, CD80 expression on TLR2^-/-^ B cells was significantly reduced by stimulation with Pam3CSK4 alone and the combination of poly I:C and Pam3CSK4 (Fig 5A). TLR3^-/-^ B cells also had significant reduction in CD80 expression after combination stimulation (Fig 5B). Proliferation was significantly reduced to the Pam3CSK4 alone and combination stimulation in TLR2^-/-^ B cells (Fig 5C). There were no significant differences in proliferation detected in the TLR3^-/-^ B cells in response to any stimulation (Fig 5D). TNFα production, which was significantly increased in wild type B cells after combination poly I:C and Pam3CSK4 stimulation, was reduced in both the TLR2^-/-^ and TLR3^-/-^ B cells (Fig 5E and 5F). Similar results were also found for other surface receptors and cytokines (S6 and S7 Figs).

For positive controls we used LPS, which signals through TLR4, and CpG, which signals through TLR9 (Fig 5C). TLR4 is the only other TLR besides TLR3 which utilizes the TRIF pathway, although only partially. Although both knockout B cells responded to LPS similar to the wild type, there was a significant drop in proliferation in the TLR2^-/-^ B cells, however TLR2 has been reported to enhance responses to LPS [37]. TLR9 is an intracellular receptor that is highly expressed by murine B cells, there were no differences in response between wild type and knockout B cells tested.

### Poly I:C and Pam3CSK4 enhance B cell induced activation of CD4^+^ T cells

**Fig 6:**
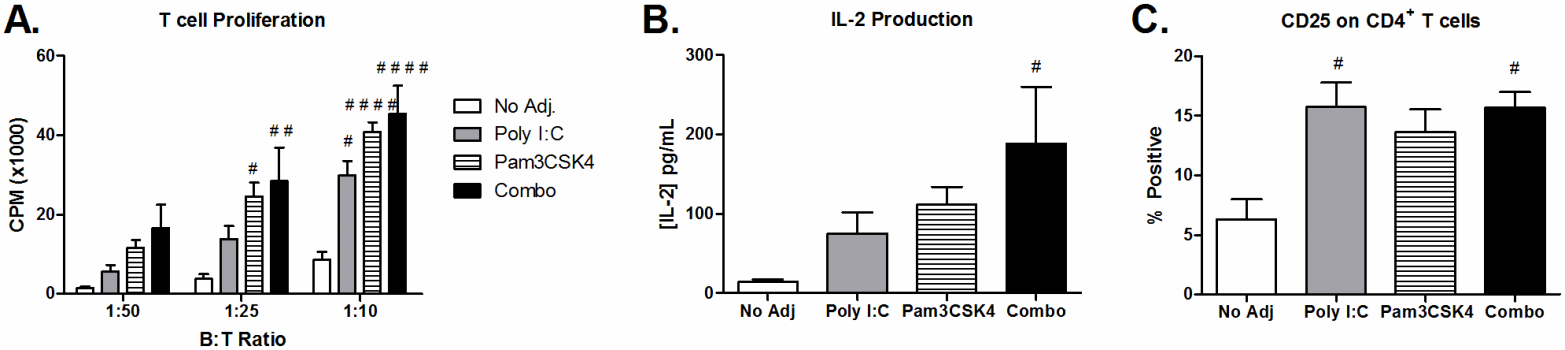
Allogeneic T cell response is increased in B cells stimulated with poly I:C and Pam3CSK4. Purified B cells from C57BL6 mice and stimulated with poly I:C (25 μg/mL), Pam3CSK4 (1 μg/mL) or a combination of both with T-cell-dependent co-stimulation provided by anti-CD40 and anti-Ig. After 24 hours, B cells were inactivated by mitomycin C, and resuspended at various concentrations. B cells were co-cultured with 100,000 CD4^+^ T cells isolated from BALB/c mice at ratios 1:10 (10,000 B cells), 1:25 (4,000 B cells) or 1:50 (2,000 B cells). (A) Proliferation was measured after 3 days by [^3^H]-TdR incorporation. Separate co-cultures were setup in parallel to detect IL-2 production (B) in supernatant as well as expression of CD25 (C) on CD4^+^ T cells after 3 days. Results are shown as the average ± SEM of 5 individual B cell preparations from 5 independent experiments. Statistics performed by ANOVA with Tukey post-test: “#” indicates significance relative to untreated.

Stimulation of B cells with poly I:C and Pam3CSK4 resulted in increased expression of several co-stimulatory molecules involved in T cell activation, such as CD80, CD86, CD40 and MHC class II. Therefore, we tested whether poly I:C and Pam3CSK4 activated B cells could more efficiently stimulate CD4^+^ T cell proliferation using an allogeneic model. We found that B cells activated with poly I:C and Pam3CSK4 were most efficient at inducing CD4^+^ T cell proliferation (Fig 6A). CD4^+^ T cell activation was confirmed by increased levels of IL-2 detected in co-culture supernatants (Fig 6B) and expression of high affinity IL-2 receptor CD25 on CD4^+^ T cells (Fig 6C).

### B cell differentiation into antibody producing plasma cells is enhanced by poly I:C and Pam3CSK4

**Fig 7:**
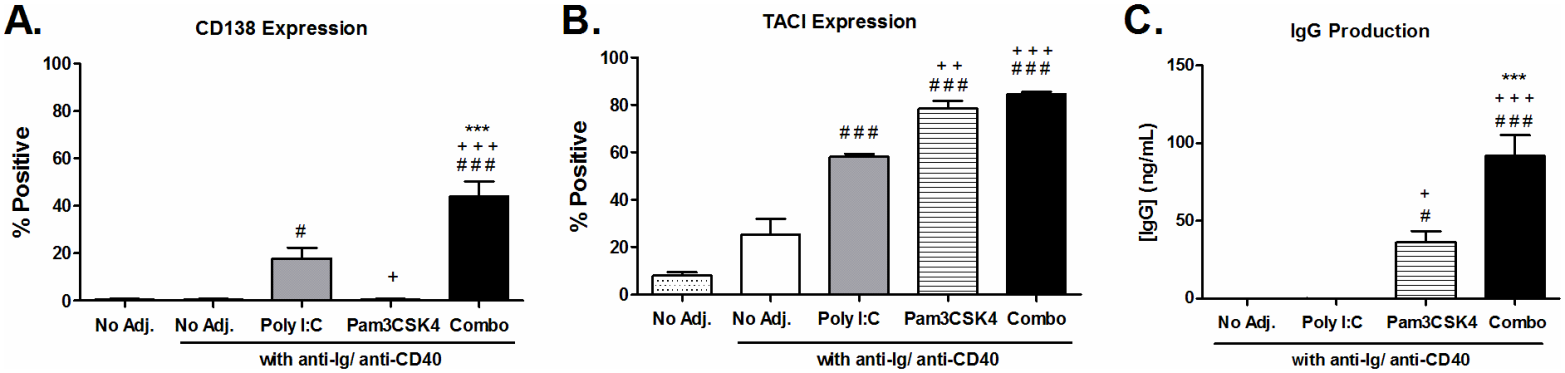
Antibody production and plasma cell marker expression are increased on B cells following T-cell-dependent stimulation with poly I:C and Pam3CSK4. Purified B cells from C57BL/6 mice were stimulated with poly I:C (25 μg/mL), Pam3CSK4 (1 μg/mL) or a combination of both with T-cell-dependent co-stimulation provided by anti-CD40 and anti-Ig. Surface expression of (A) CD138 (n = 7) and (B) TACI (n = 4) were determined by flow cytometry after 24 hours incubation. IgG was detected in supernatants harvested after 4 days of incubation by ELISA (n = 5). Results are shown as the average ± SEM of individual B cell preparations as indicated, data is pooled from two to three independent experiments. Statistics performed by ANOVA with Tukey post-test: “#” indicates significance relative to untreated, “+” indicates significance relative to poly I:C and “*” indicates significance relative to Pam3CSK4.

To determine if poly I:C and PamCSK4 could promote B cell differentiation into ASC, we looked for markers of plasma cell differentiation on B cells that had been stimulated for 24 hours with poly I:C and/ or Pam3CSK4. The combination treatment resulted in the highest levels of surface receptors associated with plasma cell differentiation, CD138 and TACI (CD267) (Fig 7A and 7B). Supernatants harvested after 4 days of culture also had high levels of IgG present in the B cells stimulated with the combination, indicating polyclonal activation of B cells. Notably, the levels of IgG in the supernatants of B cells stimulated with poly I:C and/ or Pam3CSK4 was below the limits of detection.

### Protein vaccines adjuvanted with poly I:C and Pam3CSK4 produce highest levels of antibodies in vivo

**Fig 8:**
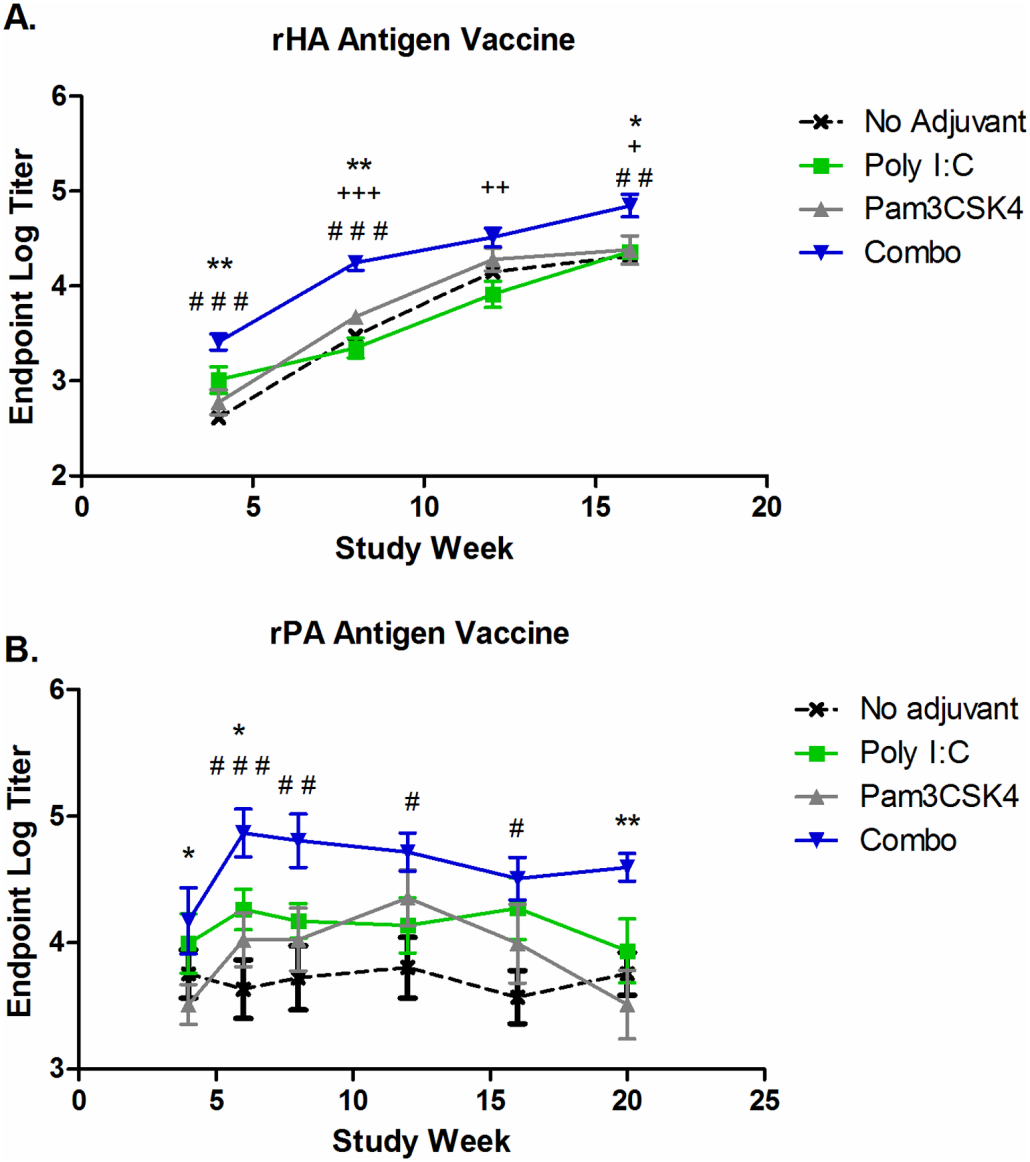
Poly I:C and Pam3CSK4 adjuvant combination enhance antibody production by influenza and anthrax vaccines *in vivo*. (A) CD-1 mice (n = 8) were vaccinated once with influenza recombinant hemagglutinin antigen (rHA; 0.5ug) formulated in DepoVax with no adjuvant, poly I:C (1 μg), Pam3CSK4 (1 μg) or the combination of both. (B) CD-1 mice (n = 8) were vaccinated once with anthrax recombinant protective antigen (rPA; 1 μg) formulated in DepoVax with no adjuvant, poly I:C (1 μg), Pam3CSK4 (1 μg), or the combination of both. For A and B, mice were bled on the indicated weeks after immunization and antigen-specific antibodies detected in serum by direct ELISA. Results are shown as endpoint titre ± SEM and are each representative of two independent experiments. Statistics by 2-way ANOVA with Bonferroni post-test comparing the combination to: “#” compared to no adjuvant, “+” compared to poly I:C; “*” compared to Pam3CSK4. No differences were detected between other groups.

Having demonstrated that poly I:C and Pam3CSK4 could augment T-cell-dependent B cell activation *in vitro*, resulting in enhanced function and differentiation into ASC, we sought to determine if these agonists could be used to boost antibody response to vaccination *in vivo*. We evaluated the poly I:C/ Pam3CSK4 adjuvant system using two different antigens: recombinant hemagglutinin (rHA) H5N1 for influenza and recombinant protective antigen (rPA) for anthrax. The vaccines were formulated using the DPX platform and contained no adjuvant, poly I:C at 1 μg dose, Pam3CSK4 at 1 μg dose or the combination of both adjuvants. The adjuvant dose was selected based on preliminary *in vivo* dose-response screening in which each adjuvant was separately dosed in DPX with a rPA antigen (S8 Fig). We choose the minimal doses that enhanced immune responses over non-adjuvanted vaccine. These experiments were conducted in the outbred CD-1 mouse strain in order to increase the translational relevancy of the findings. Naïve CD-1 mice received a single vaccination and antigen-specific antibody titers were monitored in the serum every four weeks post immunization by ELISA using Protein A for detection. Endpoint titers for each group are shown in Fig 8. For both the influenza and anthrax vaccine, the combination of poly I:C and Pam3CSK4 resulted in rapid induction of antibodies that were at significantly higher levels than the non-adjuvanted vaccine or vaccines with containing single adjuvants.

## Discussion

Development of novel vaccines that can induce strong and robust antibody responses with minimal immunizations is critical to providing effective protection from influenza and anthrax infection. One aspect of this development process is the identification of adjuvant systems that can boost antibody-mediated immune responses towards vaccination. In this study we identified an adjuvant system comprised of two TLR ligands, poly I:C and Pam3CSK4, that had a potent effect on B cell activation *in vitro* and could enhance antigen-specific antibody production towards vaccination with DPX formulated influenza and anthrax vaccines *in vivo*. To our knowledge, this is the first extensive characterization of the effects of poly I:C and Pam3CSK4 stimulation on B cells and documents enhanced B cell activation in response to stimulation with a combination of these agonists. This particular combination of agonists has translational relevancy as both the receptors TLR3 and TLR2 are expressed in similar levels in both mouse and human naïve B cells [12].

B cell activation *in vitro* was measured by changes in surface receptor expression, cytokine production (after 24 hours) and proliferation (after 3 days). Previous reports have shown that poly I:C and Pam3CSK4 can each stimulate B cell activation at low levels [25, 26]. Our study of various doses of both poly I:C and Pam3CSK4 clearly demonstrate that these agonists can provide activation signals to B cells when used at optimal concentrations. Each agonist resulted in a characteristic activation profile of the B cells, suggesting non-redundancy in signaling pathways. Ablation of response in TLR3^-/-^ and TLR2^-/-^ B cells demonstrated that poly I:C and Pam3CSK4 were signaling through these receptors. The additive or synergistic effect on activation may be attributed to differential signaling pathways utilized by each receptor, as TLR3 signals through the adaptor protein TRIF while TLR1/2 signals through the adaptor protein MyD88 [8]. The majority of studies on intrinsic TLR signaling on B cell activation have focused on TLR4 and TLR9 since these receptors are strongly expressed on murine B cells [12]. Our results suggest that other TLR ligands could have equally strong effects despite low expression, and that the B cell response may be regulated by requiring more than one ligand. In particular, TLR4 is not expressed on naïve human B cells as it is on murine B cells [12], our results may suggest that combining TLR4 agonists with other TLR agonists may increase the translational relevancy of these adjuvants.

Stimulation with the poly I:C and Pam3CSK4 agonist combination induced increased expression of CD80 and CD86, which are co-receptors that are important for antigen-presentation to CD4^+^ T cells. The expression of CD80 never reached the levels of the other surface receptors evaluated, but it has been reported that expression of CD80 peaks 48–72 hours after upregulation [38]. Ppoly I:C and Pam3CSK4 stimulated B cells were most efficient at promoting allogeneic CD4^+^ T cell proliferation. An interesting finding was the increased expression of CD25, the high affinity IL-2 receptor, on B cells in response to poly I:C and Pam3CSK4 stimulation. While the B cells alone did not produce detectable amounts of IL-2, CD4^+^ T cells in co-culture with B cells did. This may indicate a mechanism through which B cell activation is amplified during a T-cell-dependent antigen response. Of note, CD25^+^ B cell isolated from murine splenocytes have been demonstrated by others to have enhanced ability to stimulate T cells *in vitro* [39].

Poly I:C and Pam3CSK4 induced a significant increase in IL-6, TNF-α and CXCL10 secretion by B cells. Both IL-6 and TNF-α are important cytokines involved in promoting B cell survival and proliferation [40]. CXCL10 is a chemokine recognized by CXCR3, an important chemokine receptor involved in migration of activated T cells during Th1-type immune responses [41]. Poly I:C is known to induce expression of CXCL10 in other cell types [35]. Expression of CXCL10 by activated B cells could therefore promote B and T cell interactions by recruiting activated T cells expressing CXCR3.

Differentiation of B cells into antibody producing plasma cells *in vitro* was enhanced by poly I:C and Pam3CSK4 stimulation in combination with T-cell-dependent stimulation. It is noteworthy that stimulation with poly I:C alone induced CD138 expression whereas Pam3CSK4 did not, and yet Pam3CSK4 alone induced IgG secretion whereas poly I:C did not. In both cases, the combination of poly I:C and Pam3CSK4 induced the highest levels of both CD138 and IgG secretion. This could be an indication that signaling pathways induced by either TLR3 or TLR1/2 are subject to cross-regulation, and stimulation through one receptor enhances the pathways induced by the other. This type of interaction has been previously observed on macrophages stimulated sequentially with poly I:C and Pam3CSK4 [29]. B cells stimulated in T-cell-independent conditions did not produce detectable antibody secretion, indicating that the effects of these adjuvants on B cells in vivo would be unlikely to result in non-specific production of antibodies if stimulation occurred in the absence of peptide presentation to the B cells.

TACI is a TNF superfamily receptor member expressed only by B lymphocytes. TACI can respond to B cell survival factors BAFF and APRIL, which are primarily produced by non-B cells in order to regulate B cell survival and differentiation during B cell maturation. Previously, TACI has been shown to be increased on murine B cells in response to TLR stimulation by LPS and CpG cells [42, 43]. TACI itself interacts with intracellular MyD88 and converges with signaling induced by TLR and CD40 to promote class switch recombination in B cells [44]. In our studies, antibody production could only be induced in T-cell-dependent activation of B cells, and was augmented by poly I:C and Pam3CSK4 combination treatment. Although it is unlikely that TACI signaling participated in this event *in vitro* since BAFF and APRIL are not produced by B cells, it is interesting to speculate that increased TACI expression promoted by poly I:C and Pam3CSK4 contributes to more efficient plasma cell differentiation *in vivo*.

TLR agonists are commonly used as vaccine adjuvants to boost antibody production; however, the contribution of direct TLR stimulation on B cells in development of antibody responses *in vivo* is not well understood [21]. Various studies using conditional knockouts for MyD88 or MHC class II expression have demonstrated that initiation of immune responses can be reliant on either B cells or DC, depending on the antigen type and availability [20, 45, 46]. Nevertheless, our *in vivo* studies using vaccines for influenza and anthrax clearly demonstrate that poly I:C and Pam3CSK4 adjuvant combination result in enhanced antigen-specific antibodies. However, we could not demonstrate that this effect was due to intrinsic TLR signaling on B cells *in vivo*. Most likely, the enhanced *in vivo* effect was due to TLR signaling induced by these agonists on both B cells and DCs. Further exploration of this would require extensive studies using knockout mice, this would be a valuable project to pursue.

B cell interaction with antigen or adjuvant in the lymph node is facilitated by phagocytes that can transport intact antigens and adjuvants from the periphery using non-degradative intracellular compartments [13, 14]. The vaccine delivery system used in this study, DepoVax^™^ (DPX), is an oil based formulation that forms a depot in vivo. Using MRI to detect iron-labeled antigens, we have demonstrated that the immune system plays an active role in taking up the vaccine and transporting the components to the lymph node [3]. In this way, DPX is unlike other vaccine platforms which are commonly either an aqueous buffer or emulsion, both of which allow vaccine components to diffuse from the vaccine site more readily (manuscript in preparation). The active uptake mechanism of DPX ensures that vaccine components are directly delivered to immune system cells. In this work, we have shown how different adjuvants or adjuvant combinations may affect immune responses induced by the vaccine, and this may be partially attributed to direct stimulation of B cells by poly I:C and Pam3CSK4 made possible using the DPX formulation.

The poly I:C and Pam3CSK4 adjuvant system induced a strong and robust antigen-specific antibody response *in vivo*. The doses used were the minimal dose required to elicit antigen-specific antibody responses *in vivo* (S8 Fig). Since this response could be induced with minimal doses of each adjuvant together, the adjuvant system is not only more cost effective, but also reduces the potential for an of adverse reaction to the vaccine. There is an urgent need to develop more effective vaccination strategies for both anthrax and influenza. A potential added benefit of using a dual adjuvant system is that these adjuvants, in particular poly I:C, are also known to induce potent T cell responses that can contribute to robust immunity [47]. The effect of poly I:C/ Pam3CSK4 adjuvant system on the kinetics of T cell response warrants further investigation.

In conclusion, we have demonstrated that poly I:C and Pam3CSK4 stimulation of B cells results in a unique activation profile and when used in combination contribute to the optimal induction of B cell activation. Poly I:C and Pam3CSK4 together are an effective adjuvant system capable of boosting antibody responses *in vivo* when administered with protein antigens and could prove to be a promising new DPX-based vaccine formulation for influenza and anthrax indications.

## Supporting information

S1 Fig Purity of B cells isolated from spleen. (A) Staining of C57BL/6 splenocyte starting populations with CD3-FITC (145-2C11), CD11c-PE (N418) and CD19-APC (1D3). (B) Staining of purified B cell populations for same markers. (C) Phenotypic analysis of purified B cells before culture staining with CD23-FITC (B3B4), B220-PE (RA3-6B2), CD5-APC (5373) and IgD-APC (11-26c).
(PDF)

S2 Fig Representative histograms for isotype controls. Purified C57BL/6 CD19^+^ B cells were stimulated with poly I:C (25 ug/mL), Pam3CSK4 (1 ug/mL) or the combination of both adjuvants for 24 hours. B cells were then analysed by flow cytometry using isotype controls (A) Armenian Hamster IgG-FITC (eBio299Arm), (B) Rat IgG2a-PE (aBR2a), (C) Rat IgG1-APC (eBRG1). Representative of at least three independent experiments.
(PDF)

S3 Fig Dosing of poly I:C and PamCSK4 in vitro on B cell response. B cells were isolated from the spleens of naïve C57BL/6 mice (n = 3) and stimulated with various concentrations of poly I:C and Pam3SK4. Expression of CD40 (A), CD80 (B) and MHC class II (C) was determined by flow cytometry after 24 hour stimulation. Dashed line indicates level of unstimulated B cells. Data are shown as average ± SEM of 3 individual B cell preparations as indicated and was collected in a single experiment(D) Proliferation of B cells was measured after 3 days incubation by [^3^H]-TdR uptake (n = 2–7). Data shown as average ± SEM of 2–7 individual B cell preparations pooled from at least 2 separated experiments. Statics by 1-way ANOVA with Dunnett’s post-test comparing each dose to unstimulated, *p<0.05, **p<0.01, ***p<0.001.
(TIF)

S4 Fig Dose response to poly I:C and Pam3CSK4 combinations in vitro. B cells were isolated from the spleens of naïve C57BL/6 mice (n = 3) and stimulated with various concentrations of poly I:C and Pam3SK4 alone and in combination for 24 hours. Expression of CD80 (A), CD40 (B), MHC class II (C) was detected by flow cytometry. Secretion of IL-6 (D) was detected by ELISA, BLD: below limit of detection. Results are shown as the average of 3 individual B cell preparations and was collected in a single experiment, the selected combination of poly I:C (25 μg/mL) and Pam3CSK4 (1 μg/mL) is bolded.
(PDF)

S5 Fig Representative histograms for B cell surface marker expression. Purified C57BL/6 CD19^+^ B cells were stimulated with poly I:C (25 ug/mL), Pam3CSK4 (1 ug/mL) or the combination of both adjuvants for 24 hours. B cells were then analysed by flow cytometry for expression of CD86, CD80, CD25, MHC class II (IA/IE), CD69 and CD40. Results from multiple experiments are summarized in Fig 1.
(PDF)

S6 Fig TLR2 knockout B cell stimulation. CD19^+^ B cells were purified from TLR2^-/-^ (n = 4) or C57BL/6 wild type (n = 4) mice and stimulated with poly I:C (25 ug/mL), Pam3CSK4 (1 ug/mL) or the combination of both adjuvants for 24 hours in (A) T-cell-independent and (B) T-cell-dependent conditions. B cells were analysed by flow cytometry for expression of CD40, CD86, MHC class II, CD25 and CD80. (C) Supernatants were analysed by ELISA for CXCL10.
(TIF)

S7 Fig TLR3 knockout B cell stimulation. CD19^+^ B cells were purified from TLR3^-/-^ (n = 5) or B6;129SF2/J wild type (n = 4) mice and stimulated with poly I:C (25 ug/mL), Pam3CSK4 (1 ug/mL) or the combination of both adjuvants for 24 hours in (A) T-cell-independent and (B) T-cell-dependent conditions. B cells were analysed by flow cytometry for expression of CD40, CD86, MHC class II, CD25 and CD80. (C) Supernatants were analysed by ELISA for IL-6. (D) Supernatants were analysed by ELISA for CXCL10.
(TIF)

S8 Fig Dosing of poly I:C and Pam3CSK4 in rPA vaccine. CD-1 mice were vaccinated with rPA antigen (2 ug) formulated with (A) poly I:C or (B) Pam3CSK4, at indicated doses, in DPX. Antigen-specific antibodies were detected in serum at 4 and 8 weeks post immunization.
(TIF)
