# Supplementary figures and images for: Combination of poly I:C and Pam3CSK4 enhances activation of B cells in vitro and boosts antibody responses to protein vaccines in vivo
Source: PLoS One. 2017 Jun 29;12(6):e0180073. doi: 10.1371/journal.pone.0180073 (PMC5491120; doi:10.1371/journal.pone.0180073)

**A.**

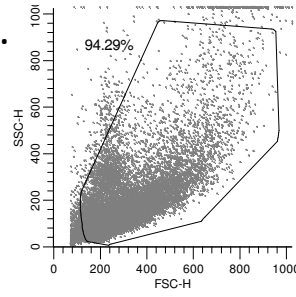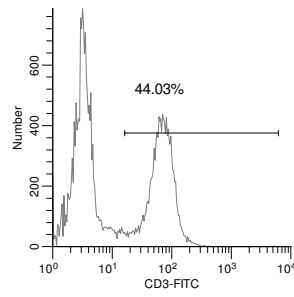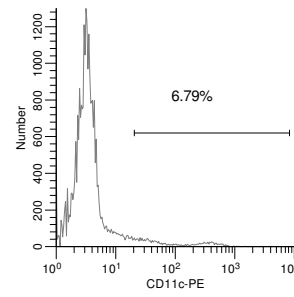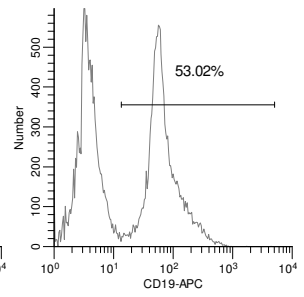

**B.**

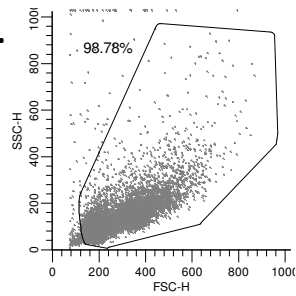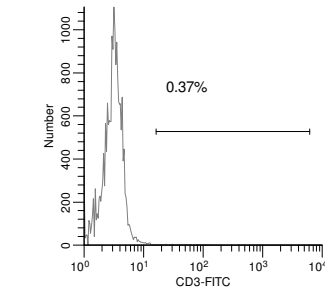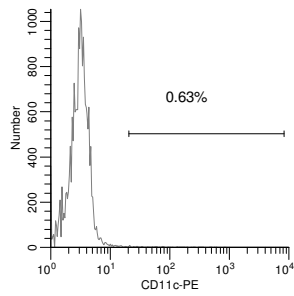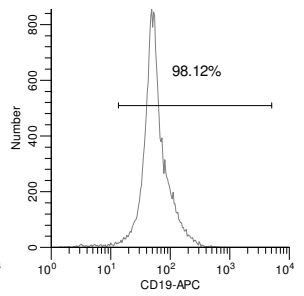

**C.**

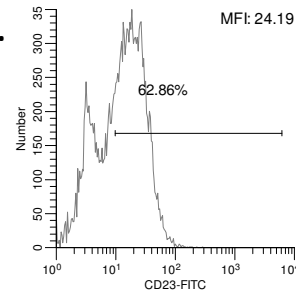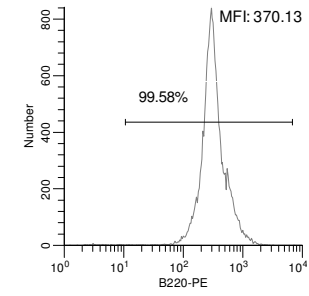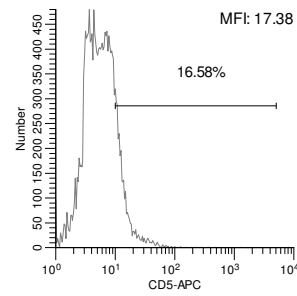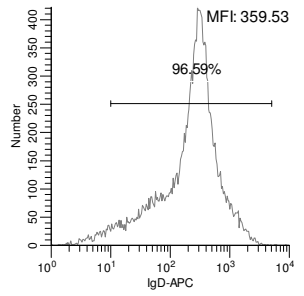

Supplement: S1 Fig — (A) Staining of C57BL/6 splenocyte starting populations with CD3-FITC (145-2C11), CD11c-PE (N418) and CD19-APC (1D3). (B) Staining of purified B cell populations for same markers. (C) Phenotypic analysis of purified B cells before culture staining with CD23-FITC (B3B4), B220-PE (RA3-6B2), CD5-APC (5373) and IgD-APC (11-26c). (PDF) [file pone.0180073.s001.pdf]

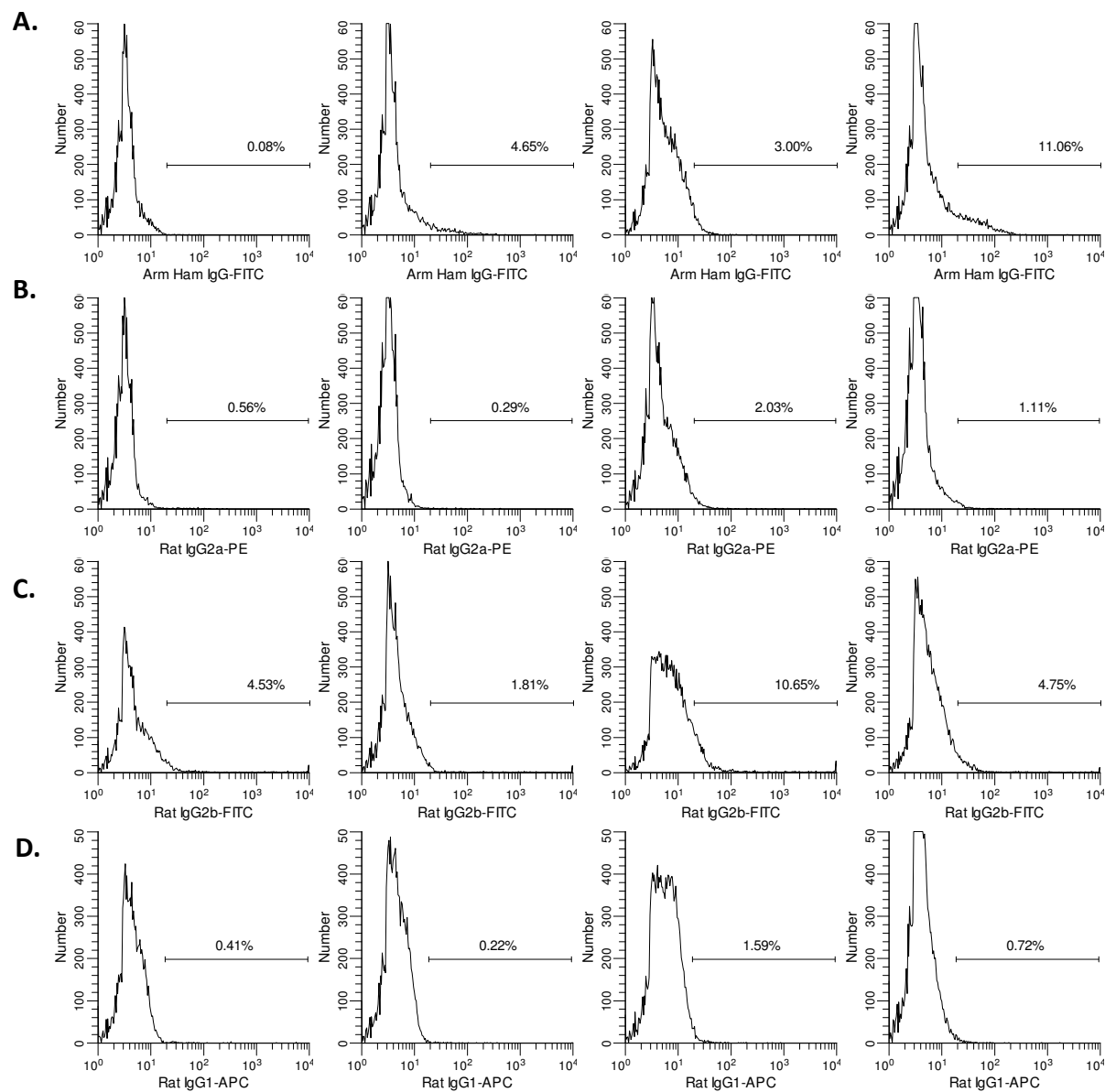

Supplement: S2 Fig — Purified C57BL/6 CD19+ B cells were stimulated with poly I:C (25 ug/mL), Pam3CSK4 (1 ug/mL) or the combination of both adjuvants for 24 hours. B cells were then analysed by flow cytometry using isotype controls (A) Armenian Hamster IgG-FITC (eBio299Arm), (B) Rat IgG2a-PE (aBR2a), (C) Rat IgG1-APC (eBRG1). Representative of at least three independent experiments. (PDF) [file pone.0180073.s002.pdf]

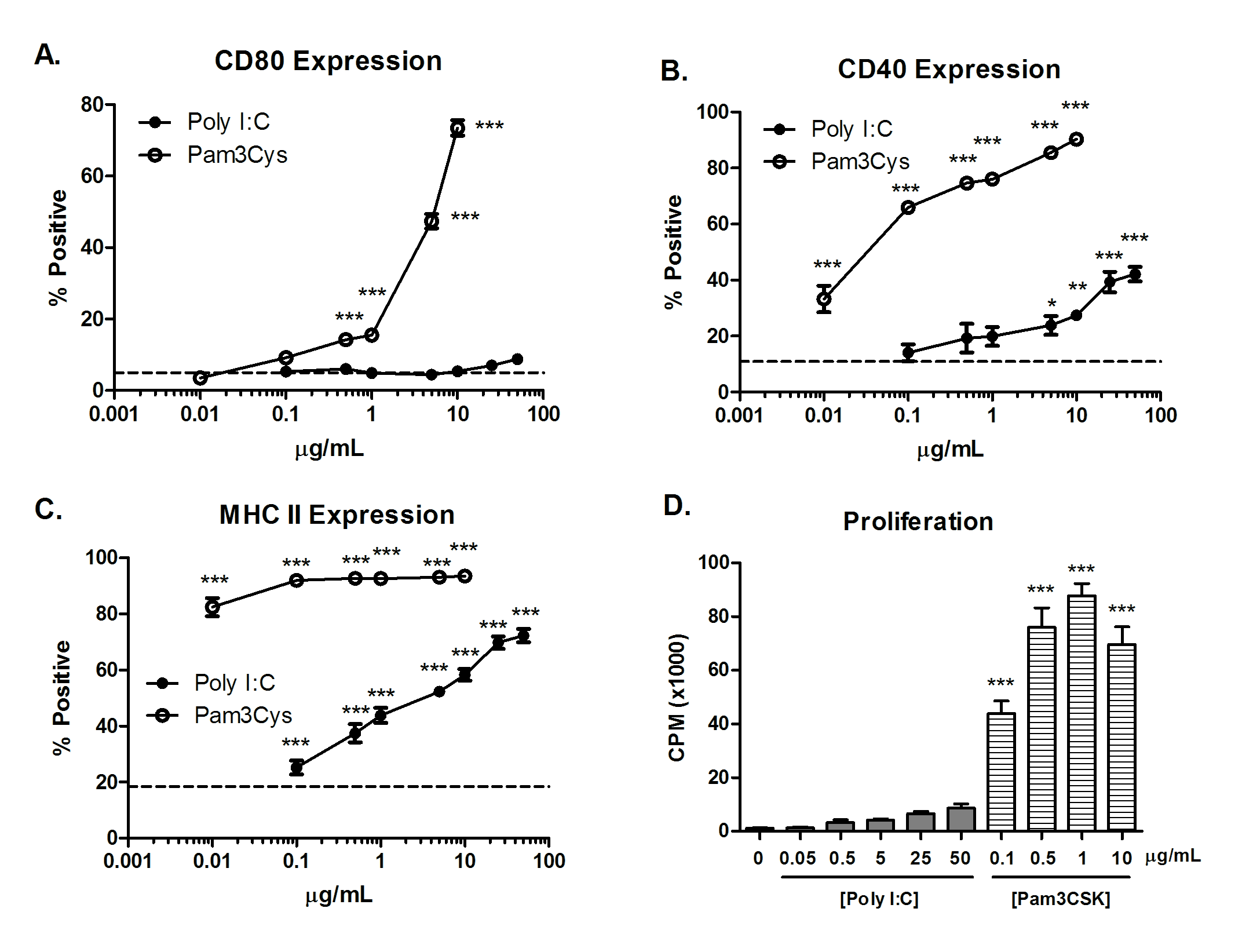

Supplement: S3 Fig — B cells were isolated from the spleens of naïve C57BL/6 mice (n = 3) and stimulated with various concentrations of poly I:C and Pam3SK4. Expression of CD40 (A), CD80 (B) and MHC class II (C) was determined by flow cytometry after 24 hour stimulation. Dashed line indicates level of unstimulated B cells. Data are shown as average ± SEM of 3 individual B cell preparations as indicated and was collected in a single experiment(D) Proliferation of B cells was measured after 3 days incubation by [3H]-TdR uptake (n = 2–7). Data shown as average ± SEM of 2–7 individual B cell preparations pooled from at least 2 separated experiments. Statics by 1-way ANOVA with Dunnett’s post-test comparing each dose to unstimulated, *p<0.05, **p<0.01, ***p<0.001. (TIF) [file pone.0180073.s003.tif]

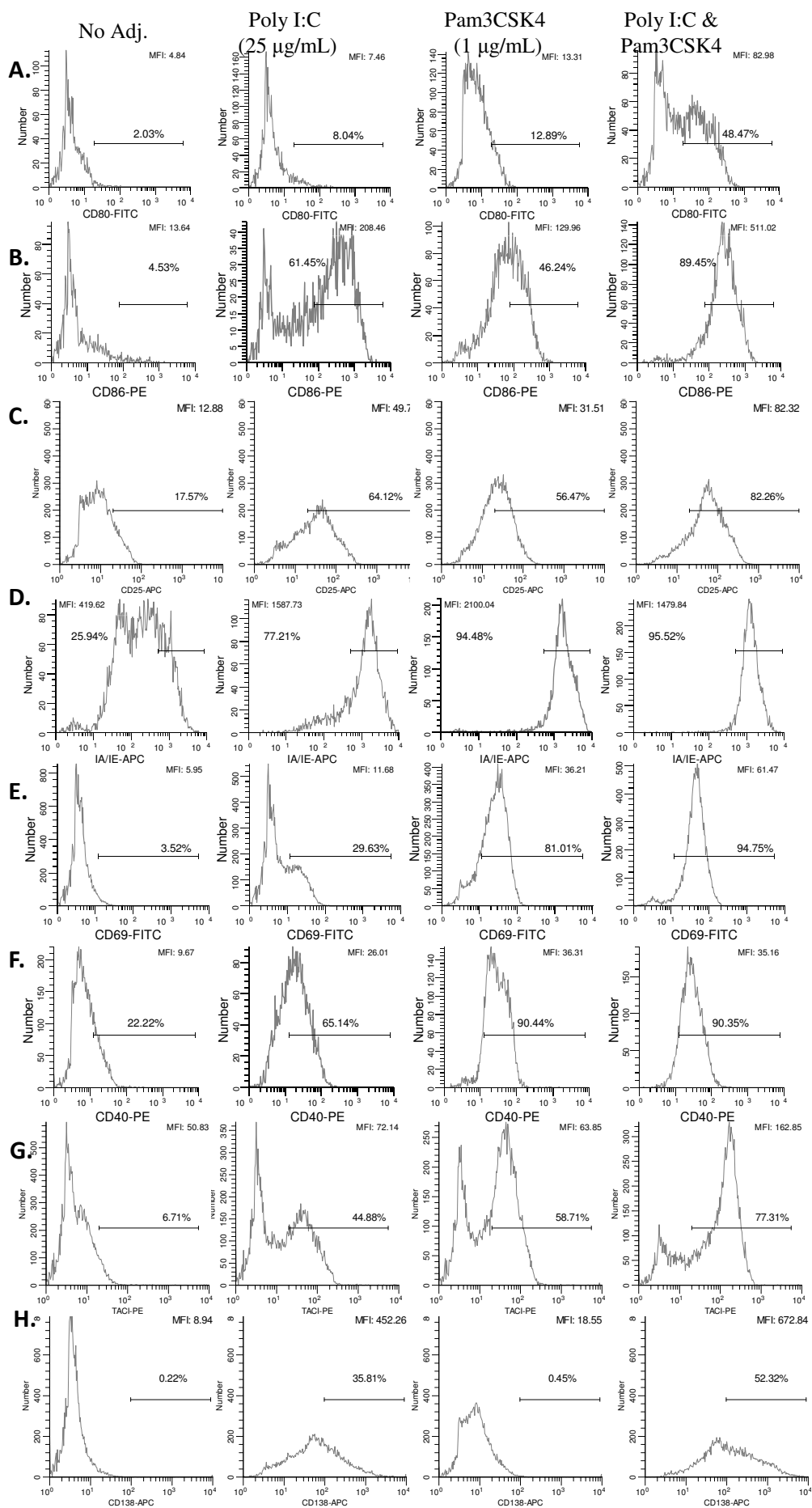

Supplement: S5 Fig — Purified C57BL/6 CD19+ B cells were stimulated with poly I:C (25 ug/mL), Pam3CSK4 (1 ug/mL) or the combination of both adjuvants for 24 hours. B cells were then analysed by flow cytometry for expression of CD86, CD80, CD25, MHC class II (IA/IE), CD69 and CD40. Results from multiple experiments are summarized in Fig 1. (PDF) [file pone.0180073.s005.pdf]

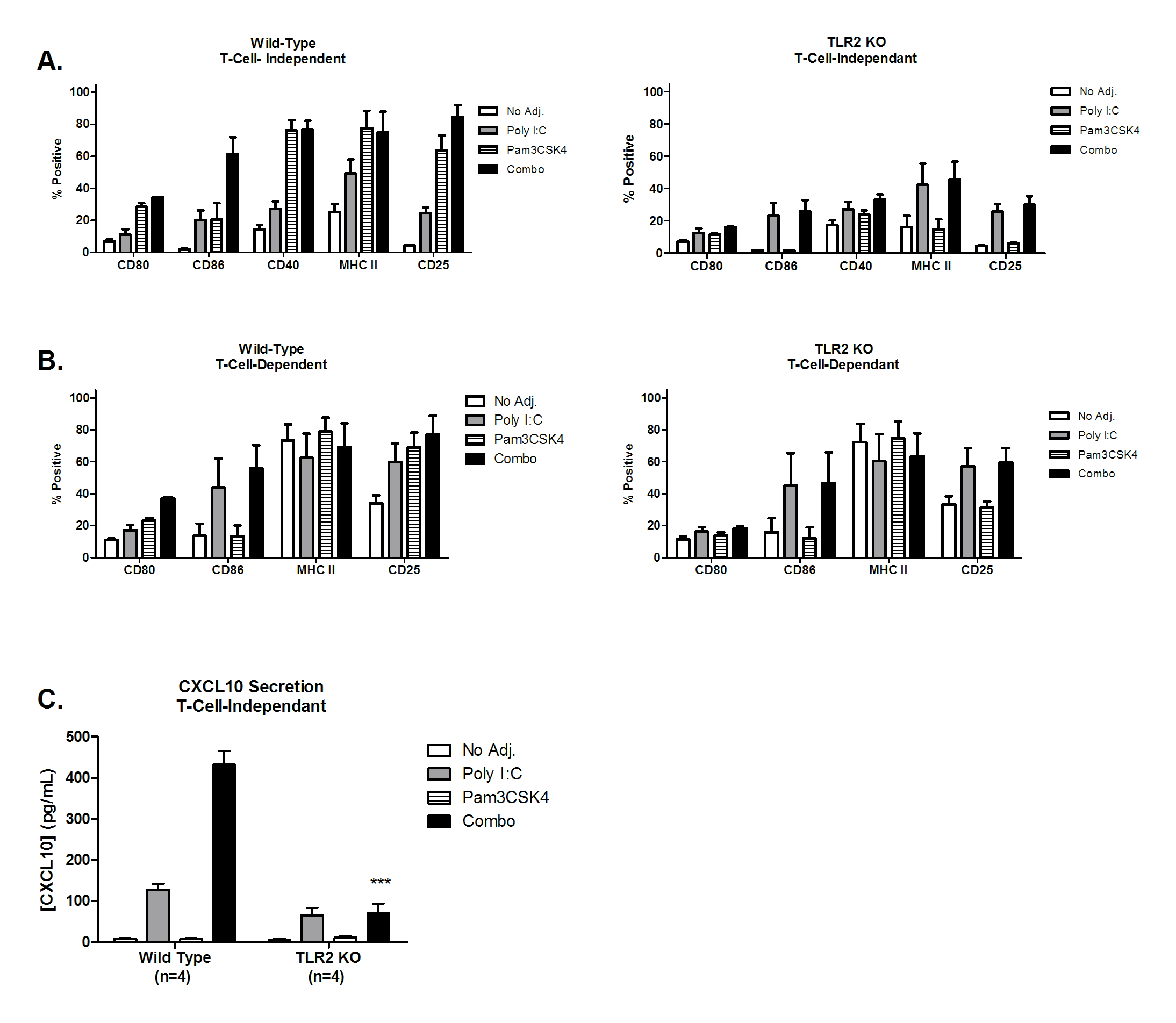

Supplement: S6 Fig — CD19+ B cells were purified from TLR2-/- (n = 4) or C57BL/6 wild type (n = 4) mice and stimulated with poly I:C (25 ug/mL), Pam3CSK4 (1 ug/mL) or the combination of both adjuvants for 24 hours in (A) T-cell-independent and (B) T-cell-dependent conditions. B cells were analysed by flow cytometry for expression of CD40, CD86, MHC class II, CD25 and CD80. (C) Supernatants were analysed by ELISA for CXCL10. (TIF) [file pone.0180073.s006.tif]

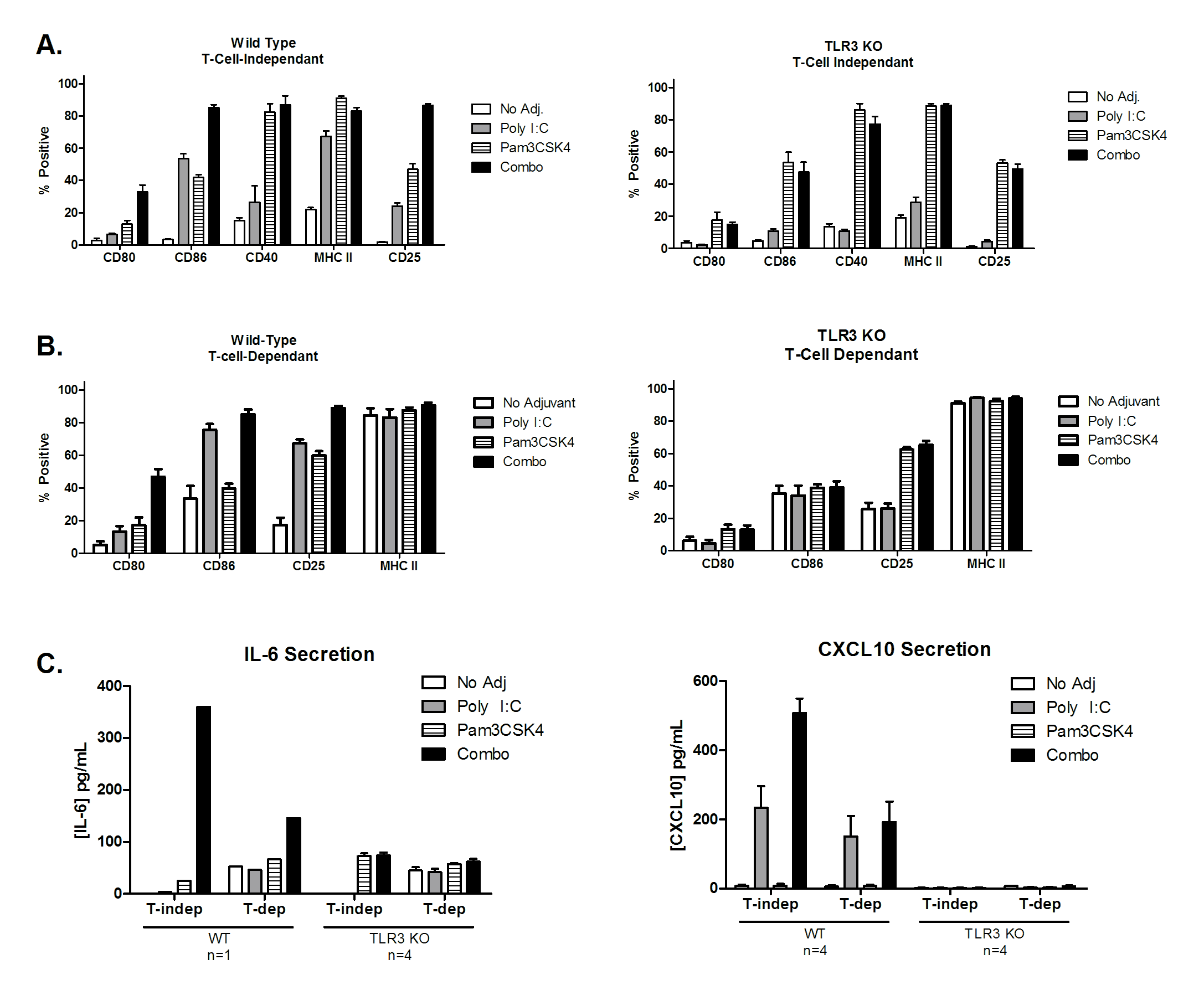

Supplement: S7 Fig — CD19+ B cells were purified from TLR3-/- (n = 5) or B6;129SF2/J wild type (n = 4) mice and stimulated with poly I:C (25 ug/mL), Pam3CSK4 (1 ug/mL) or the combination of both adjuvants for 24 hours in (A) T-cell-independent and (B) T-cell-dependent conditions. B cells were analysed by flow cytometry for expression of CD40, CD86, MHC class II, CD25 and CD80. (C) Supernatants were analysed by ELISA for IL-6. (D) Supernatants were analysed by ELISA for CXCL10. (TIF) [file pone.0180073.s007.tif]

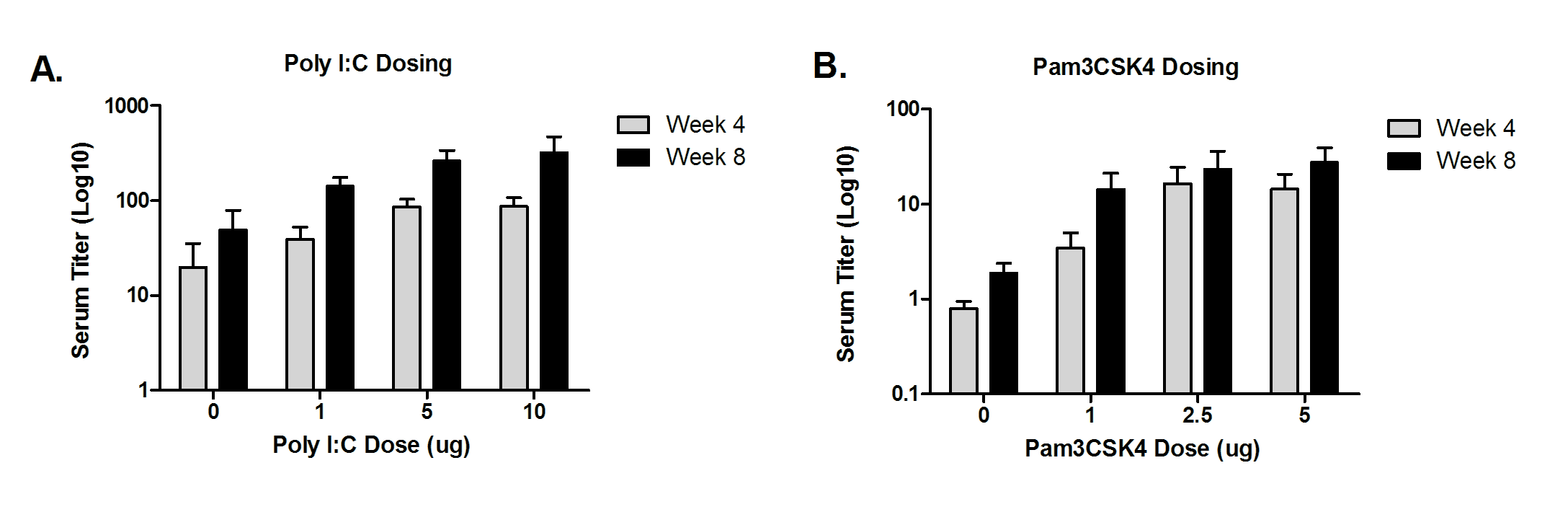

Supplement: S8 Fig — CD-1 mice were vaccinated with rPA antigen (2 ug) formulated with (A) poly I:C or (B) Pam3CSK4, at indicated doses, in DPX. Antigen-specific antibodies were detected in serum at 4 and 8 weeks post immunization. (TIF) [file pone.0180073.s008.tif]
