# Supplementary material for: Combination of poly I:C and Pam3CSK4 enhances activation of B cells in vitro and boosts antibody responses to protein vaccines in vivo
Source: PLoS One. 2017 Jun 29;12(6):e0180073. doi: 10.1371/journal.pone.0180073 (PMC5491120; doi:10.1371/journal.pone.0180073)
Supplement: S4 Fig — B cells were isolated from the spleens of naïve C57BL/6 mice (n = 3) and stimulated with various concentrations of poly I:C and Pam3SK4 alone and in combination for 24 hours. Expression of CD80 (A), CD40 (B), MHC class II (C) was detected by flow cytometry. Secretion of IL-6 (D) was detected by ELISA, BLD: below limit of detection. Results are shown as the average of 3 individual B cell preparations and was collected in a single experiment, the selected combination of poly I:C (25 μg/mL) and Pam3CSK4 (1 μg/mL) is bolded. (PDF) [file pone.0180073.s004.pdf]

(A) % CD80

Poly I:C (ug/mL) →

Pam3CysK4 (ug/mL) ↓

	0	0.1	0.5	1	5	10	25	50
**0**	4.98	5.30	6.04	4.89	4.42	5.38	7.06	8.75
**0.01**	3.48	3.38	5.99	6.16	10.40	13.49	24.09	33.31
**0.1**	9.17	12.07	18.23	24.60	34.85	40.53	50.13	52.23
**0.5**	14.25	20.79	29.79	34.41	44.13	49.87	58.07	58.60
**1**	15.49	24.52	34.85	41.35	46.32	52.43	**60.98**	59.17
**5**	47.37	47.52	51.29	50.33	61.65	61.18	66.84	64.92
**10**	73.46	74.81	73.66	78.21	59.61	67.75	56.66	64.60

(B) % CD40

Poly I:C (ug/mL) →

Pam3CysK4 (ug/mL) ↓

	0	0.1	0.5	1	5	10	25	50
**0**	11.05	14.13	19.31	19.94	23.89	27.48	39.36	42.12
**0.01**	33.28	34.04	39.08	41.03	49.40	56.11	61.98	69.70
**0.1**	65.99	73.84	77.33	79.58	76.88	79.01	76.42	80.00
**0.5**	74.60	77.86	83.50	83.15	83.37	83.50	80.69	82.51
**1**	76.05	76.05	82.27	83.65	82.56	80.93	**83.34**	82.17
**5**	85.45	85.98	87.15	83.84	91.43	83.42	81.84	81.68
**10**	90.26	90.01	88.03	89.84	90.17	88.67	79.28	79.83

(C) % MHC II

Poly I:C (ug/mL) →

Pam3CysK4 (ug/mL) ↓

	0	0.1	0.5	1	5	10	25	50
**0**	18.49	25.24	37.45	43.82	52.32	58.26	69.75	72.31
**0.01**	82.45	76.94	80.57	80.99	87.41	87.22	90.57	92.80
**0.1**	92.00	90.73	93.08	94.72	94.52	94.77	95.54	95.68
**0.5**	92.73	87.82	92.71	94.34	93.63	94.71	94.96	95.36
**1**	92.64	85.69	91.22	92.64	93.54	94.38	**95.20**	94.93
**5**	93.10	94.28	92.52	90.40	94.51	92.41	94.64	94.92
**10**	93.57	93.40	91.09	91.76	93.98	92.58	94.00	94.52

(D) IL-6 (pg/mL)

Poly I:C (ug/mL) →

Pam3CysK4 (ug/mL) ↓

	0	0.1	0.5	1	5	10	25	50
**0**		43.33	20.34	1.52	12.84	74.14	38.29	45.91
**0.01**	2.40	6.73	43.18	34.10	17.60	26.83	77.16	152.39
**0.1**	13.50	99.01	157.59	107.54	194.85	259.49	435.58	544.61
**0.5**	19.64	96.82	172.02	245.73	409.30	505.82	737.92	761.66
**1**	32.85	107.54	137.96	221.64	424.04	612.43	**823.96**	770.15
**5**	89.16	48.49	74.97	51.97	230.36	324.37	893.07	840.47
**10**	23.62	51.11	138.78	43.57	94.20	156.30	561.00	757.67

Lowest — **Highest**
